# PReS-FINAL-2157: Efficacy of canakinumab in the treatment of systemic juvenile idiopathic arthritis: a 12-week pooled post-hoc analysis

**DOI:** 10.1186/1546-0096-11-S2-P169

**Published:** 2013-12-05

**Authors:** P Quartier, H Brunner, T Constantin, S Padeh, I Calvo, M Erguven, L Goffin, M Hofer, T Kallinich, S Oliveira, Y Uziel, S Viola, K Nistala, C Wouters, K Lheritier, J Hruska, K Abrams, A Martini, N Ruperto, D Lovell

**Affiliations:** 1 Necker-Enfant Malades Hospital, Paris, France; 2 PRCSG, Cincinnati, OH, USA; 3 PRINTO-Istituto Gaslini, Genova, Italy; 4 Novartis Pharma, Basel, Switzerland; 5 Novartis Pharmaceuticals Corp, NJ, USA

## Introduction

Interleukin-1β (IL-1β) plays a key role in the pathogenesis of systemic juvenile idiopathic arthritis (SJIA). Efficacy and safety of canakinumab (CAN), a selective, fully human, anti- IL-1β monoclonal antibody, have been demonstrated in 2 phase III trials. Here we present 12-week results of a post-hoc pooled analysis.

## Objectives

To evaluate the 12-week efficacy of CAN 4 mg/kg in treatment naïve patients (pts).

## Methods

Data from the 3 trials done as part of the phase III program were pooled for this analysis. Pts aged 2-19 yrs with active SJIA were enrolled and received subcutaneous CAN 4 mg/kg or placebo. The post-hoc analysis presented here focuses on SJIA response to CAN therapy in the initial treatment period of a total of 178 CAN-naïve pts. Methodological factors precluded a comparator group, so this analysis is of a descriptive nature.

## Results

At baseline (BL), 94% of pts had intermittent spiking fever due to SJIA; and 73% were on steroids (mean dose of 0.38 mg/kg/d). In the pooled analysis (N = 178), by Week 2 evidence of profound clinical benefit was observed (Table 1) with 20% of pts even achieving inactive disease.

**Table 1:** Percentage of patients with adapted JIA ACR (aacr) response and inactive disease

CAN, N = 178						
**N(%)**	**Aacr30**	**Aacr50**	**Aacr70**	**Aacr90**	**Aacr100**	**Inactive disease**

**Week 2**	142 (80%)	125 (70%)	102 (57%)	65 (37%)	38 (21%)	36 (20%)

**Week 12**	125 (70%)	122 (69%)	108 (61%)	87 (49%)	54 (30%)	50 (28%)

Data from missing patients not shown; aacr response = ACR response level plus absence of fever

The median CRP level of 158 mg/L at BL decreased by a median of 82% and 94% by weeks 2 and 12, respectively. Rapid improvements were also observed in the number of active joints. The median number of active joints decreased from 10 at BL to 2.5 at Week 2 and 0 at Week 12. Similarly, for joints with limitation of motion, median values decreased from 9 at BL to 2.5 and 1 at Week 2 and 12, respectively. While 94% pts had fever due to SJIA at BL, only 13% at Week 2 and 2% at Week 12 had fever. Notably, CAN therapy resulted in marked improvement in patient reported outcomes: parent/patient assessment of pain (0-100 mm, VAS) decreased from a mean of 67 mm at BL to 22 mm at Week 2 and 11 mm at Week 12. The median CHAQ disability score decreased from 1.8 at BL to 0.6 at Week 2 and 0.3 at Week 12. Between BL and Week 12, the median physicians' global assessment of SJIA activity (0-100 mm, VAS) decreased from 70 mm to 3 mm and the parents'/patients' assessment of overall well-being improved from 63 mm to 4.5 mm.

## Conclusion

Based on this post-hoc analysis, response of the SJIA patients studied for the phase III program of CAN showed a rapid and clinically important improvement of their disease by Week 12 of therapy, with an aacr 50 or higher responses reached by the majority of the CAN-naïve pts within 2 weeks after the initial CAN dose.

## Disclosure of interest

P. Quartier Grant/Research Support from: Abbvie, Chugai-Roche, Novartis, Pfizer, Consultant for: Abbott/Abbvie, BMS, Chugai-Roche, Novartis, Pfizer, Servier, SOBI, Speakers Bureau: Chugai-Roche, Novartis, Pfizer, H. Brunner Consultant for: Novartis, Genentech, Medimmune, EMD, Serono, AMS, Pfizer, UCB, Jannsen, Speakers Bureau: Genentech, T. Constantin: None declared., S. Padeh: None declared., I. Calvo: None declared., M. Erguven: None declared., L. Goffin: None declared., M. Hofer Grant/Research Support from: Novartis, T. Kallinich Grant/Research Support from: Novartis, Speakers Bureau: Roche, Novartis, ALK, S. Oliveira Grant/Research Support from: Novartis, Y. Uziel Consultant for: Novartis, S. Viola Consultant for: To Gaslini Hospital: Abbott, Astrazeneca, BMS, Centocor Research & Development, Eli Lilly and Company, "Francesco Angelini", Glaxo Smith & Kline, Italfarmaco, Novartis, Pfizer Inc., Roche, Sanofi Aventis, Schwarz Biosciences gmbh, Xoma, Wyeth Pharmaceuticals Inc., K. Nistala: None declared., C. Wouters Grant/Research Support from: Novartis Belgium, Roche Belgium, GSK immunoinflammation USA, K. Lheritier Employee of: Novartis, J. Hruska Employee of: Novartis, K. Abrams Shareholder of: Novartis, Employee of: Novartis, A. Martini Consultant for: To Gaslini Hospital: Abbott, Astrazeneca, BMS, Centocor Research & Development, Eli Lilly and Company, "Francesco Angelini", Glaxo Smith & Kline, Italfarmaco, Novartis, Pfizer Inc., Roche, Sanofi Aventis, Schwarz Biosciences gmbh, Xoma, Wyeth Pharmaceuticals Inc., Speakers Bureau: Bristol-Myers Squibb, Novartis, Astrazeneca, Glaxo Smith and Kline, N. Ruperto Grant/Research Support from: To Gaslini Hospital: Abbott, Astrazeneca, BMS, Centocor Research & Development, Eli Lilly and Company, "Francesco Angelini", Glaxo Smith & Kline, Italfarmaco, Novartis, Pfizer Inc., Roche, Sanofi Aventis, Schwarz Biosciences gmbh, Xoma, Wyeth Pharmaceuticals Inc., Speakers Bureau: Astrazeneca, Bristol Myers and Squibb, Janssen Biologics B.V., Roche, Wyeth/Pfizer, D. Lovell Grant/Research Support from: NIH, Consultant for: Astra-Zeneca, Centocor, Jansen, Wyeth, Amgen, Bristol-Meyers Squibb, Abbott, Pfizer, Regeneron, Hoffman La-Roche, Novartis, Genentech, Speakers Bureau: Genentech, Roche.

